# Interaction of sulfasalazine with outer surface of boron-nitride nanotube as a drug carrier in aqueous solution: insights from quantum mechanics and Monte Carlo simulation

**DOI:** 10.1186/s13065-023-01088-w

**Published:** 2023-11-28

**Authors:** Sepideh Ketabi, Saba Shalmashi, Sara Hallajian

**Affiliations:** 1 Department of Chemistry, Faculty of Pharmaceutical Chemistry, Tehran Medical Sciences, Islamic Azad University, Tehran, Iran; 2 Active Pharmaceutical Ingredients Research (APIRC), Tehran Medical Sciences, Islamic Azad University, Tehran, Iran; 3 Department of Organic Chemistry, Faculty of Pharmaceutical Chemistry, Tehran Medical Sciences, Islamic Azad University, Tehran, Iran

**Keywords:** SSZ, Free energy, DFT, Monte Carlo simulation

## Abstract

The improvement of the solubility of sulfasalazine in physiological media was the major aim of this study. Accordingly, BNNT inspected as a notable candidate for the carriage of this drug in aqueous media. For this purpose, four possible interactions of two tautomer of sulfasalazine with (9,0) boron-nitride nanotube were considered in aqueous media. The compounds were optimized in gas phase using density functional calculations. Solvation free energies and association free energies of the optimized structures were then studied by Monte Carlo simulation and perturbation method in water environment. Outcomes of quantum mechanical calculations presented that interaction of keto form of sulfasalazine produce the most stable complexes with boron-nitride nanotube in gas phase. Simulation results revealed that electrostatic interactions play a vital role in the intermolecular interaction energies after binding of drug and nanotube in aqueous solution. Results of association free energy calculations indicated that complexes of both two sulfasalazine tautomers (keto and enol) and nanotube were stable in solution. Computed solvation free energies in water showed that the interaction with boron-nitride nanotube significantly improved the solubility of sulfasalazine, which could improve its in vivo bioavailability.

**Supplementary Information:**

The online version contains supplementary material available at 10.1186/s13065-023-01088-w.

## Introduction

Sulfasalazine (SSZ) is a nonsteroidal anti-inflammatory and anti-rheumatic drug that is extensively used for the treatment of ulcerative colitis [1], rheumatoid arthritis and Crohn’s disease [2, 3]. However, the side effects of excessive use of SSZ are hemopoietin system disorder, severe allergy, and antibiotic resistance [4, 5]. Hence, the concentration of SSZ in the body should be controlled to avoid the side effects.

Oral use of SSZ is mostly recommended in high doses. In this way, only one third of the drug is absorbed in the small intestine, and the rest is transferred to the large intestine, where it undergoes reduction by bacterial reductases and produces sulfapyridine and mesalazine [6, 7]. Since SSZ its unstable, poorly soluble in aqueous solution, and has low membrane permeability, its oral bioavailability is very low [7]. Accordingly, different drug delivery systems have been proposed to enhance the bioavailability and reduce drug dosage and side effects [8–10].

In recent years, a lot of research has been done to improve new drug delivery systems.

SSZ amphiphilic derivative has been synthesized by interacting with polyethylene glycol to produce self-assembled nanostructure [9]. By forming core-shell nanostructures with hydrophilic polyethylene glycol on the surface and hydrophobic SSZ in the core, an aqueous solution of SSZ–polyethylene glycol has been obtained.

Most recently loading of SSZ in cyclodextrin metal organic framework (MOF) has been studied as drug delivery system [10]. The influence of metal organic framework on important medicinal properties of SSZ such as dissolution and membrane permeability has been demonstrated. MOF-loaded SSZ enhanced drug release.

One of the most important aspects of nanotechnology is the improvement of new and actual drug delivery systems [11–15]. In recent years, drug delivery performance of the hexakis dimer as a new carrier for doxorubicin (DOX) has been evaluated by density functional theory (DFT) calculations [16]. The results have indicated that the adsorption of the DOX drug on the hexakis dimer spontaneously proceeded.

Possibility of a stable interaction between favipiravir drug molecule and BN-doped C60 (CBN) heterofullerene have been investigated using DFT/B3LYP calculations [17]. It has concluded that CBN heterofullerene can be used as a delivery tool to decrease harmful effects of the favipiravir drug. In another study the adsorption behavior of oxadiazole molecule on pristine, B-, Al- and Ga-doped C60 fullerenes have been evaluated by DFT calculations [18]. Adsorption energies for the interactions between oxadiazole and the nano carriers have increased compared to pristine C60. It has been defined that Al-doped C60 fullerenes are preferred candidates for drug adsorption. Capability of pristine, B-, Al-, and Ga-doped CNTs as drug delivery system for 5-fluorouracil (5-FU) drug molecule have also investigated by quantum mechanical calculations [19]. According to the results, Al and Ga dopants have induced an increase in the adsorption capacity of CNT. However, overall results suggest that Al-doped CNT can be used as a delivery tool for cancer treatment.

The preferences of nanostructures include high specific surface area, chemical activity and increased permeability [20, 21]. In recent years SSZ has been loaded on the surface of Au doped B12N12 nanocluster to design an efficient drug carrier [22]. The interaction of SSZ drug with Au decorated B12N12 nanoclusters has been investigated using B3LYP, B3PW91, and PBE / DFT calculations. The negative values of binding energies stated the possibility of the interactions. Modification of the electronic properties of B12N12 nanoclusters after the adsorption of SSZ has introduced them as efficient biosensors.

Recently, the adsorption of SSZ on the B16N16 nanocluster has been investigated using DFT/PBE1PBE1/6-311G (d, p) calculations [23]. Then doped Al and Si atoms instead of a B atom in the B16N16 have been studied to propose an efficient sensor for SSZ. The adsorption energies of stable compounds of SSZ with B16N16, SiB15N16, and AlB15N16 was reported to be − 24.58, − 30.39, and − 53.43 kcal mol^−1^, respectively. To evaluate the performance of nanoclusters in the body fluids, the adsorption of SZZ on the B16N16, AlB15N16, and SiB15N16 in water has been calculated applying the polarizable continuum model. The energy values in aqueous solution have shown that nanostructures and their complexes with SSZ are stable in the water and can be used as carriers.

Among various nanomaterials, nanotubes are appropriate contenders for drug carriers. Their special feature is the apparent inner and outer surfaces for adsorption of drugs. Nanotubes can enter cells and the cell nuclei [24–29]. Furthermore, their non-toxic behavior makes them potential candidate for biomedical applications [14, 30]. Monte Carlo simulation study of the interaction of carbon nanotubes (CNTs) with β-Alanine and Histidine in aqueous solution have been revealed that adsorption of these amino acids can improve solvation of CNTs [31]. Another research has also indicated that the interaction with carnosine increases the solvation of CNT and thus reduces its toxicity. Calculated complexation free energies have confirmed the stability of CNT–carnosine systems in aqueous media, and ultimately confirm the potential applicability of CNT in medicinal chemistry [32]. CNTs have also been investigated as carriers for three alkylating agent anti-cancer drugs (Chlorambucil, Cyclophosphamide and Melphalan) by molecular simulation study in aqueous media [33]. The use of CNTs has been confirmed to carry Chlorambucil to target cells.

Structure of boron nitride nanotube (BNNT) is similar to CNT, so it has similar properties [34]. Nevertheless, BNNTs have better efficiency compared to CNTs.

The polarity of the B-N bond makes BNNTs better potential candidate for surface interactions, and the strength of B-N bond makes them stable at high temperatures, resistant to oxidation, high thermal conductivity and low electric constant [35, 36]. For example, BNNTs are more effective for hydrogen storage compared to CNTs because polar B–N bonds interact more strongly with hydrogen [36]. BNNTs have also indicated higher water transport properties than CNTs [37].

Since BNNTs are non-toxic due to their chemical inertness and structural stability, they are more applicable as drug delivery systems [38]. Ciofani et al. [39, 40] have approved the biocompatibility of BNNTs. After the injection of BNNTs in rabbits, they did not show any significant negative effects for the in vivo study. BNNTs have also shown a non-cytotoxic effect after testing on human cells, which confirms the biocompatibility of these nanotubes [41]. BNNTs have been used for in vitro experiments and have transport DNA oligomers into cells without toxicity [34]. These studies showed that BNNTs may have potential applications in biological systems. For instance, quantum mechanical investigation of the interactions of BNNTs and azomethine has been revealed that binding to BNNTs did not affect the therapeutic agent complex [42]. In our previous study, comparing the predicted properties of cisplatin anticancer drug complexes with BNNT and CNT, confirmed that BNNT can act as a more effective drug carrier. Encapsulation of cisplatin into CNT and BNNT in water have been investigated by quantum mechanical calculations and Monte Carlo simulation [43]. The obtained results have revealed that the solvation free energies of BNNT-drug complexes were larger than those of CNTs. The results pointed out that BNNTs have a great potential for use in nanomedicine.

As mentioned, SSZ is metabolized in the biological system before reaching the colon, and its metabolic products have the most side effects. To reduce metabolism, loading SSZ on nanotubes can be effective. In addition, SSZ is a poorly water-soluble drug, and it is therefore not well absorbed after oral administration. Therefore, in this work, SSZ was considered, and the application of BNNT as a drug delivery system for SSZ was investigated. The possible bindings were examined in gas phase by density functional study. Afterward interactions of SSZ and BNNT in aqueous solution, and solvation free energies of the SSZ-BNNT complexes were determined by Monte Carlo simulation. Then molecular dynamics (MD) simulation method was also implemented to reveal the dynamic features of drug transportation by BNNT.

## Computational method

In this research, the ability of BNNT to transport SSZ molecule, and the solubility of its compounds in aqueous media have been investigated. The critical concern of this research was to investigate the interaction of drug (SSZ) and BNNT in aqueous solution, and determine the binding and hydration energies of their structures. The research comprised two parts: Quantum Mechanical calculation (QM) and Monte Carlo simulation (MC). In QM part, the structures were optimized in gas phase, and then the related binding energies were computed. In the second part, MC, solvation free energies and association free energies of the compounds were determined in aqueous solution.

### QM

**Fig. 1:**
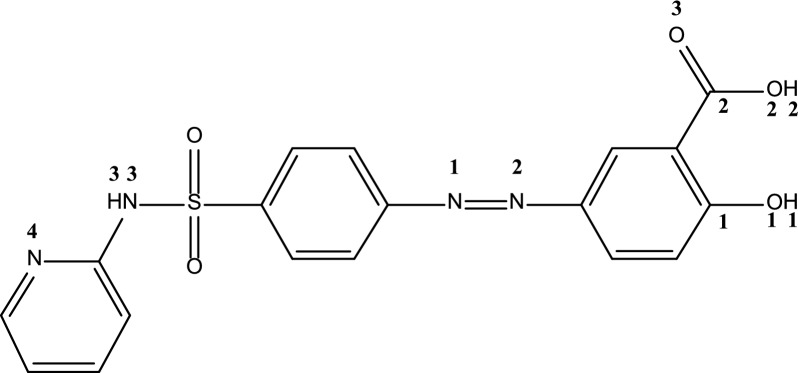
SSZ (enol form)

Zigzag (9, 0) BNNT with 198 atoms (B_99_N_99_), length of 22.63 Å and radius of 3.67 Å was evaluated as a drug carrier in this study. Two keto and enol forms of SSZ (C_18_O_5_N_4_SH_14_) were considered to interact with BNNT. The enol form of SSZ has been shown in Fig. 1, and OH of phenyl ring can be converted to an isomeric C=O group in keto form. SSZ molecule contains three different sites to interact with BNNT: pyridine ring, sulfonamide, and carbonyl group. The interactions of SSZ and nanotube were considered from these different sites: interaction of SSZ–keto from sulfonamide, from carbonyl via two vertical directions (ver1 & ver2) and from non-protonated pyridine ring, interaction of SSZ–enol from sulfonamide, from carbonyl via two vertical directions (ver1 & ver2). Since earlier research [44] has shown that the ionization constant of the pyridinium N atom of SSZ is 0.6, therefore, it is not protonated upon interaction with BNNT.

Optimization of BNNT, drug (keto and enol forms) and drug-BNNT complexes was organized by DFT/B3LYP [45–47] method and AUG-CC-pVQZ basis set [48] for S and 6– 31 G* [49, 50] basis set for the other atoms. The AIM computations were carried out using the AIM2000 program [51, 52].

For large systems such as nanostructures, weak and long-range van der Waals interactions play a significant role in their stability and reactivity, and DFT-D functionals are more convenient to these systems. The Grimme's DFT-D2 functional [53] adds an additional term to the DFT total energy to explanation of dispersion forces. We have performed quantum mechanical calculations with DFT-D model (wB97XD functional) and compared with B3LYP results in previous research [32]. The outcomes of the two methods had the same trend and confirmed the findings with plain B3LYP. Despite of insufficient description of dispersion in B3LYP, it is the popular functional used in the NTs [19, 54, 55] and it produces suitable results with less computational time. In fact, the QM structures were applied as the initial structures for MC calculations, and our major concerns were to evaluate the interaction of drug with BNNT in aqueous solution by using MC simulation method. To simulate the interactions in aqueous solution, a Lennard–Jones potential function was implemented (in addition to Coulomb potential). Therefore, the weak van der Waals interactions have been also considered in our study to compute binding energies in aqueous media accurately.

Both ends of the nanotube were saturated with hydrogen to make an accurate calculation for the infinite nanotube. Vibrational analysis was done on the optimized compounds and the characteristics of stationary points were investigated for SSZ, BNNT and SSZ–BNNT complexes. The imaginary vibration frequency was not found in the calculations, which confirms the stability of the structures. GAMESS-US quantum chemistry package [56] was implemented for QM computations.

The optimized structures of SSZ, BNNT and SSZ–BNNT complexes were applied in the MC simulation. Diluted aqueous solutions of these complexes were modeled for the simulation. Atomic partial charges extracted from QM part were applied in MC steps.

### MC

In this part the possibility of the interaction of the desired drug with BNNT in water media was investigated. In MC simulations. Canonical ensemble and Metropolis sampling [57] were employed. A cubic box with an edge of 50 Å containing about 4000 H_2_O molecules and a density of 0.993 g/cm^3^ at 298 K, [58, 59] was employed in each simulation. Dilute solutions of the SSZ and SSZ-BNNT complexes were implemented.

In every simulation cycle, a randomly selected H_2_O molecule was translated within ± 0.13 Å and rotated within ± 10° utilizing Periodic boundary conditions with acceptance of 50% for the generated configurations. Each run involved10^7^ configurations, which were extended to decrease statistical error.

The total potential energy of a solution includes intermolecular (solvent–solvent and solute–solvent interactions) and intramolecular interaction energies. The intermolecular energies include van der Waals and electrostatic energies.

To calculate H_2_O–H_2_O intermolecular interaction energies, Transferable Intermolecular Potential function (TIP3) [60, 61] with parameters q, A and C for electrostatic, repulsive and attractive van der Waals interactions was used. To calculate solute–H_2_O interaction energies, Lennard–Jones potential (with parameter ε and σ) and Coulomb potential for electrostatic interactions (with parameter q) were applied. Lennard–Jones parameters for atoms in BNNT [62]and SSZ [63] are organized in Table 1. The site–site LJ parameters were determined using Lorenz-Berthelot combining rule [64]. As mentioned earlier, atomic charges, q, were calculated in the previous QM section.

**Table 1:** LJ parameters

Site	ε, kcal mol^−1^	σ, Å
C	0.080	3.500
N (NT)	0.203	3.215
B (NT)	0.453	3.380
S	0.250	3.550
N	0.170	3.250
H	0.018	0.094
H on C	0.050	2.500

Free energy differences were calculated by Free Energy Perturbation (FEP) theory using Zwanzig equation [65]:

1 $$\Delta {\text{G}}_{{{\text{A}} \to {\text{B}})}} = - {\text{RT}}\;{\text{Ln}} < {\text{exp}} - \left( {{\text{E}}_{{\text{B}}} - {\text{E}}_{{\text{A}}} } \right)/{\text{RT > }}_{{\text{A}}}$$

Solvation free energy (ΔG_solv_ (A)) is the free energy change process in which a species is transferred from a gaseous state to a solution. Practically, the solvation free energy is determined using the perturbation method in such a way that a species disappears in the gas and solution phases:

2 $${\Delta {\text{G}}_{{{\text{solv}}({\text{A}})}} = \Delta {\text{G}}_{{{\text{gas}}({\text{A}} \to {0}})} - \Delta {\text{G}}_{{{\text{sol}}({\text{A}} \to {0}})}}$$

Association free energies of the interaction of SSZ and BNNT were calculated using appropriate thermodynamic cycle. By considering Eq. 2 and thermodynamic cycle, aassociation free energies (∆G_ass (SSZ-BNNT)_) were calculated by:

3 $$\Delta {\text{G}}_{{{\text{ass}}({\text{SSZ }} - {\text{BNNT}})}} = \Delta {\text{G}}_{{{\text{sol}}({\text{SSZ}} \to {0}})} + \, \Delta {\text{G}}_{{{\text{sol}}({\text{BNNT}} \to {0}})} - \, \Delta {\text{G}}_{{{\text{sol}}({\text{SSZ }}{-}{\text{BNNT}} \to {0}})}$$

∆G_sol (SSZ →0)_, ∆G_sol (BNNT→0)_ and ∆G_sol (SSZ –BNNT→0)_ denote free energy differences of disappearing SSZ, BNNT and SSZ–BNNT complexes in solution.

### MD

The conformations of four SSZ (keto)–BNNT complexes were equilibrated using molecular dynamics simulation (MD). All MD simulations performed with A NVT canonical ensemble and a time step of 0.2 fs for 10 ns at 298 K. The structures were placed in the center of a cubic box of size 50 Å. Then the system was equilibrated in TIP3 water medium. Intra and intermolecular interactions within the systems were defined using Universal force field [63]. Berendsen thermostat [66] was adopted to retain simulation temperature. A cutoff distance of 9.5 Å for electrostatic interactions was engaged, and van der Waals interactions were also truncated at 9.5 Å. MD simulations of present study were accomplished using LAMMPS [67] molecular dynamics software.

It is worth mentioning that some conformational changes occur on the time scale of only tens of nanoseconds, which can compromise MC simulations. In this regard, a theoretical strategy to select promising conformations from MC simulation is crucial to determine the theoretical accuracy. So, great computational effort is necessary to carry out this kind of simulation. In general, as many conformations are generated in a hybrid conventional Quantum-Mechanics/Molecular Mechanics Molecular Dynamics (QM/MM–MD) and Quantum-Mechanics/Molecular Mechanics Monte Carlo (QM/MM–MC), the number of QM calculations required is too high. Therefore, a large computational effort is necessary for this kind of simulation. There are methods of conformation selection to reduce computational cost such as the methods of statistical inefficiency [68, 69], clustering [70], and PCA (Principal Component Analysis) [71, 72]. Recently, to reduce the number of frames of MC simulations to rationalize the theoretical findings and without loss of the relevant information from the simulation, new methods based on the statistical inefficiency, Principal Component Analysis and wavelet analysis for selecting MC conformations had been developed [73, 74]. The method is a selection structure technique from MC conformations based on the wavelet transform (WT) which is named OWSCA (Optimal Wavelet Signal Compression algorithm [73–76]. WT is a relatively new mathematical tool, that can simultaneously provide frequency and time information, and signal representation. The WT for signal analysis has the advantage of the abundance of the developed wavelet functions families for each type of compound and system. This contributes to increase the efficiency of wavelets to select relevant structures.

The differences of OWSCA with other methods such as clustering method for selecting structures is that using OWSCA method considerably decreases the number of conformations without losing important information. In this study, the clustering method was to select and optimize solute structures (BNNT and BNNT–SSZ complexes). Moreover, in all MC simulations, the importance sampling method [57] was used to overcome statistical inefficiency. However the.

OWSCA method for the selection of structures can be used effectively in various types of systems and organic compounds in solution [74]. Therefore, the OWSCA method, as a more appropriate selection method, can be preferably applied in further research.

## Results and discussion

### QM

The purpose of this section was to evaluate the binding affinity of BNNT and SSZ in the gas phase. Since SSZ exists in two keto and enol form, the interaction of both tautomers of this drug with BNNT was considered in this study.

A systematic analysis was performed on several directions of SSZ adsorption on the BNNT surface. As previously mentioned, SSZ and BNNT interactions were investigated trough all three sites (pyridine ring, sulfonamide, and carbonyl group). After the optimization, the main directions were established arbitrating from the values of binding energies. The results of the quantum mechanical calculations on the studied models in gas phase are presented in Table 2. Binding energy (E_b_) were calculated according to the expression:

4 $${\text{E}}_{{\text{b}}} ( {{\text{BSSE}}\;{\text{corrected) }}}  = {\text{E}}\left( {{\text{BNNT}} - {\text{SSZ}}} \right) -  {{\text{ [E}}\left( {{\text{SSZ}}} \right) + {\text{E}}\left( {{\text{BNNT}}} \right)} ] + {\text{BSSE}}$$

where E (BNN-SSZ), E (SSZ) and E (BNNT)] determined the energy of optimized single structures of drug–BNNT, drug and BNNT. Calculations were executed with counterpoise corrections for basis set superposition error (BSSE) [77].

**Table 2:** QM results in gas phase

Species	Dipole moment (Debye)	Energy (kcal /mol)	Binding energy (kcal /mol)
BNNT	13.72	− 4,957,999.32	–
SSZ (enol form)	5.24	− 1,058,804.12	–
SSZ (keto form)	7.91	− 1,058,794.61	–
SSZ (enol)–BNNT-via carbonyl-ver1	13.52	− 6,016,807.22	− 3.78
SSZ (enol)–BNNT-via carbonyl-ver2	7.71	− 6,016,802.45	− 2.27
SSZ (enol)–BNNT-via sulfonamide	14.96	− 6,016,802.45	0.99
SSZ (keto)–BNNT-via carbonyl-ver1	21.78	− 6,016,812.11	− 18.18
SSZ (keto)–BNNT-via carbonyl-ver2	22.68	− 6,016,818.58	− 24.64
SSZ (keto)–BNNT-via sulfonamide	21.27	− 6,016,810.92	− 16.99
SSZ (keto)–BNNT-via pyridine	14.49	− 6,016,799.32	− 5.39

The results revealed that the binding energy for the adsorption of the keto form of SSZ on the outer surface of BNNT is greater than that of the enol form. The reason can be deduced accounting the higher stability of enol form due to the intramolecular hydrogen bonding between the enol hydrogen and neighboring carbonyl oxygen. Therefore, the tendency of the enol form of the drug molecule to interact with the nanotube is less than that of keto form. In fact, SSZ exists only in the stable enol form, and during the interaction with BNNT, an intermediate keto-enol structure of SSZ is produced. Then effective and stable binding of drug and BNNT is established.

As can be seen in Table 2, the binding energies of SSZ (keto form) in complexes via carbonyl 1, 2 orientation, sulfonamide and pyridine, were − 18.18, − 24.64, − 16.99 and − 5.39 kcal/mol respectively. Therefore, the interaction of keto form of SSZ from carbonyl with BNNT is preferred in gas phase.

**Fig. 2:**
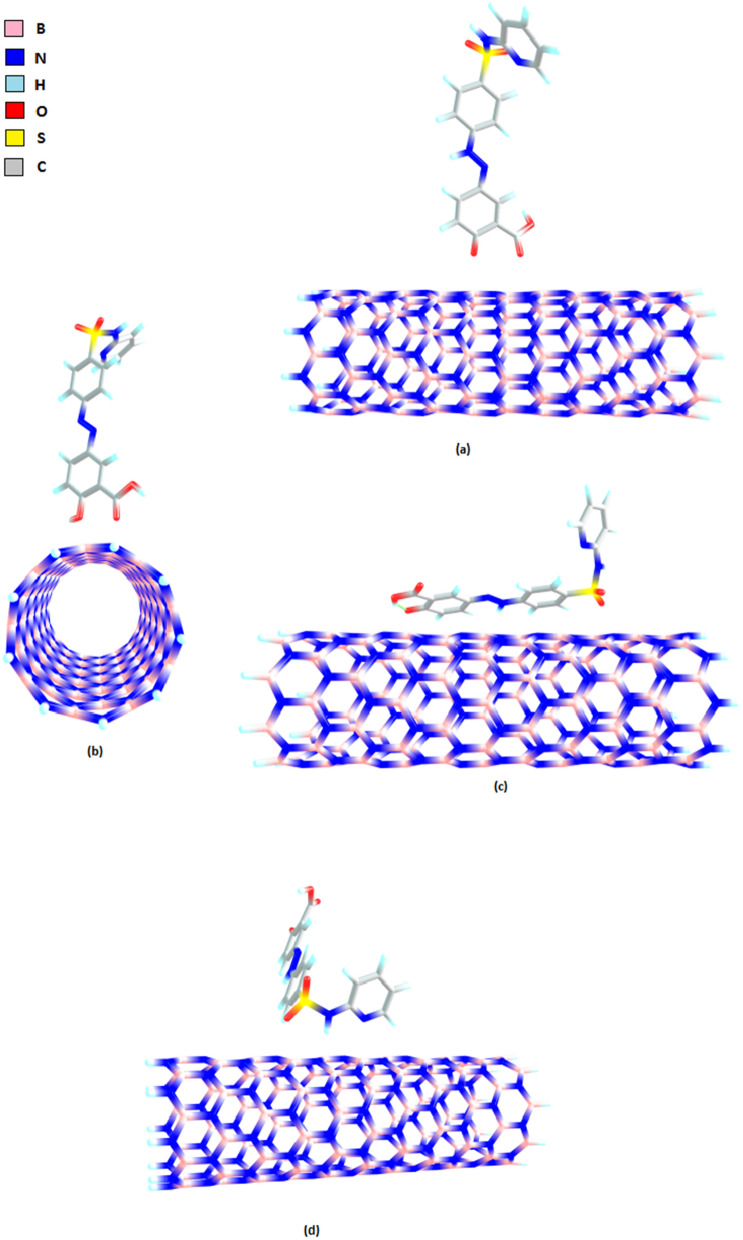
Optimized structures of SSZ–BNNT via: **a** carbonyl (vertical 1) **b** carbonyl (vertical 2) **c** sulfonamide **d** pyridinic nitrogen

Our quantitative results are comparable to another research that recently determined the adsorption energies of SSZ and B_16_N_16_ nano cluster by DFT (PBE1PBE1/6-311G (d, p) method [23]. The adsorption energies of SSZ and B_16_N_16_ nano cluster through pyridine ring, sulfonamide and carbonyl group have been about − 24.58, − 9.32, and − 16.00 kcal mol^−1^and, so the interaction of SSZ and B16N16 from pyridine ring has been preferred. Furthermore, in the complexation of glutathione transferase and SSZ, binding to the pyridinic nitrogen is preferred over the sulfonamidic N [78]. However, pyridinic nitrogen was not the strongest binding site in our study due to the steric hindrance of the nanotube. In addition, π-π stacking was one of the important components of the interaction of SSZ–BNNT in this study. Therefore, binding of SSZ through sulfonamide was even stronger than that of pyridine ring. The optimized structures of SSZ—BNNT complexes are displayed in Fig. 2.

The study of the complex formation between gold and silver with sulfasalazine has indicated that the drug interacts with metals through carboxyl groups [79]. The complex characterization has been performed by combining theoretical and experimental data. The IR, NMR and mass spectroscopic data provided essential information on the starting geometries subjected to DFT optimization.

Another research applied an effective approach for the application of nanocarriers by loading SSZ on the surface of Au doped B12N12 nanocluster [22]. The interactions have been analyzed using different DFT functionals (B3LYP, B3PW91, and PBE). All the possible orientations (pyridine, sulfonamide, and carbonyl groups) have been investigated to obtain the most interacting site of SSZ molecule. Calculated binding energies have indicated that pyridine bound more strongly (adsorption energy of 0.79 eV) than other functionalities. The difference with our research is due to the nature of nanocarrier, which is Au (doped B-N cluster) and the charge transfer occurred from lone pair of nitrogen in pyridine ring to the Au. While, the interaction site in our research was the B-N π-bond. The details of the type of molecular interactions will be discussed in the following sections. Also, the ability of SSZ to interact with B_16_N_16_, B_15_GeN_16_ and B_15_SiN_16_ nanocages has been investigated by DFT study [80] and Anti-inflammatory effects on cardiovascular disease and breast cancer have been compared with pure SSZ. The calculation results have revealed that the preferred interaction of SSZ over considered nanocages has occurred through its pyridine ring. Moreover, the adsorption of SSZ via sulfonamide showed the lowest binding energy from SO_2_ group, which is somewhat compatible with our results. The pyridine ring was not the strongest site of interaction in our results due to the steric hindrance of BNNT compared to the nanocages. In addition, the nitrogen lone pair electrons of pyrimidine occupy the SP^2^ orbital which are less available for sharing than the P orbital.

The adsorption of SSZ on the outer surfaces of pure and Ca doped C_20_ have been investigated in water media by CAM-B3LYP functional [81]. It has been revealed that SSZ adsorption on the surface of Ca doped C_20_ occurred through a weak binding energy via electrostatic interaction (non-covalent). Molecular docking study confirmed that the interaction of SSZ with Ca-doped C_20_ can improve the inhibition of proinflammatory cytokines in comparison with the pure drug. Although the nano-carrier used is different from the current study, the results specified the efficiency of the application of nano-carriers that can improve the pharmacokinetic behavior and oral bioavailability of SSZ.

The use of BN hetero-fullerene as a drug delivery system for favipiravir drug has been studied by DFT/ B3LYP method to clarify the possible interaction mechanism of adsorption process of drug molecule [17]. It has been proven that the interaction between favipiravir and BN hetero-fullerene occurred through the O atom of the drug with the B atom of the carrier with an energy of − 23.95 kcal/mol. In another study, hydroxylated fullerene has been applied as a drug carrier for SSZ [82]. The success of SSZ loading has been confirmed by thermal gravimetric analysis (TGA), Fourier transform infrared (FTIR) and Raman analysis. Furthermore, the effect of nano-carrier application on drug performance has also been investigated by comparing the antibacterial activity of SSZ-loaded fullerene with pure drug. The results approved that application of nano-carrier improves the performance of SSZ drug.

**Fig. 3:**
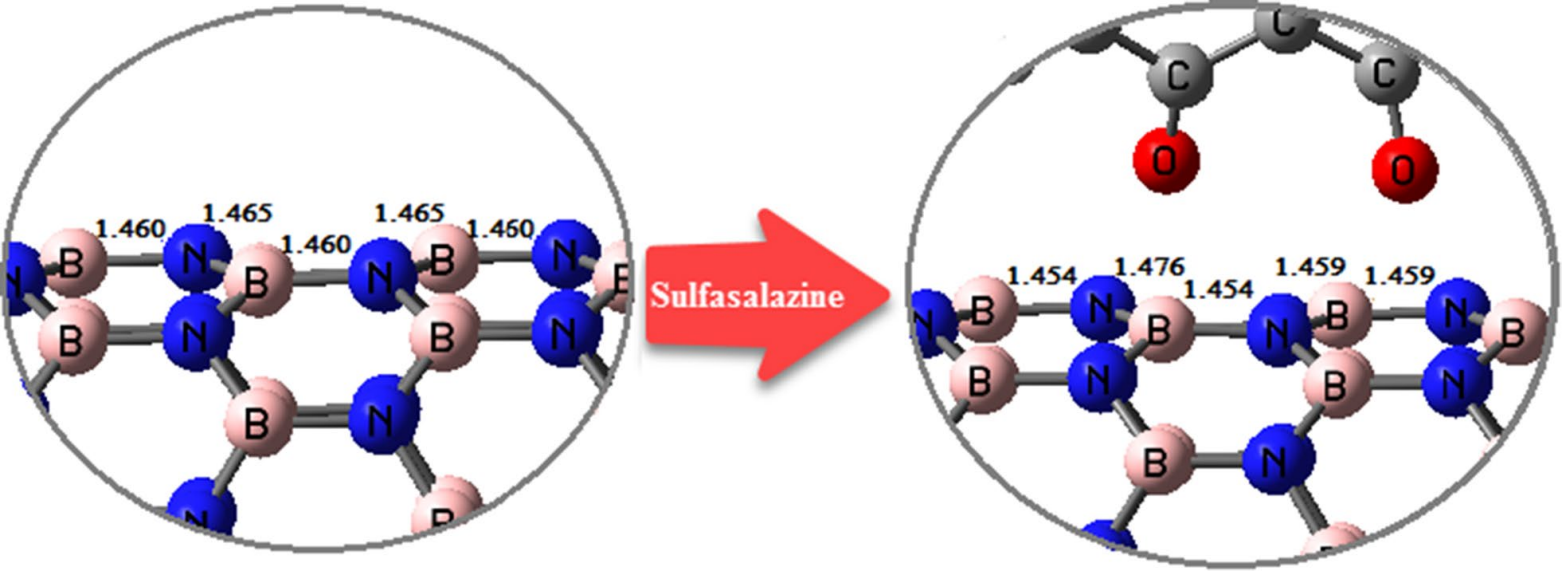
Changes of B-N bond length of nano-carrier (BNNT) through the interaction with SSZ

The structural changes of BNNT before and after the interactions with SSZ are represented in Fig. 3. The B-N bonds length for the optimized BNNT were in the range of 1.450–1.460 Å, which is consistent with other experimental (1.452 Å) and theoretical (1.455 Å) reports [83–85]. As expected, the B-N bond lengths at the binding site increased through interactions with SSZ.

The selected bond lengths of SSZ and BNNT-SSZ complexes were organized in Additional file 1: Table S1 (provided in the supporting information). According to Additional file 1: Table S1, the increase in the bond lengths occurred at drug interaction sites. For instance, during the interaction of SSZ with BNNT through the carbonyl group, the CO bond length increased due to the interaction of CO with BNNT.

Molecular electrostatic potential (MEP) and electrostatic surface potential (ESP) were also applied to evaluate the active sites the SSZ that have potential interaction sites with BNNT. The MEP surface is known as a method of mapping electrostatic potential (ESP) onto the iso-electron density surface [86]. MEP shows simultaneously molecular shape, size and ESP regions depending on color classification. The MEP graphs show the electron-rich (high electron density) or electron-poor (low electron density) side of the structures and represent the sides for nucleophilic or electrophilic interactions. The positive electrostatic potential region is often colored as blue in the MEP map whereas the negative electrostatic potential region is colored as red. The interaction of a drug molecule with the carrier usually modifies the electron density in the system and therefore an interaction can be inferred from this modification [87].

**Fig. 4:**
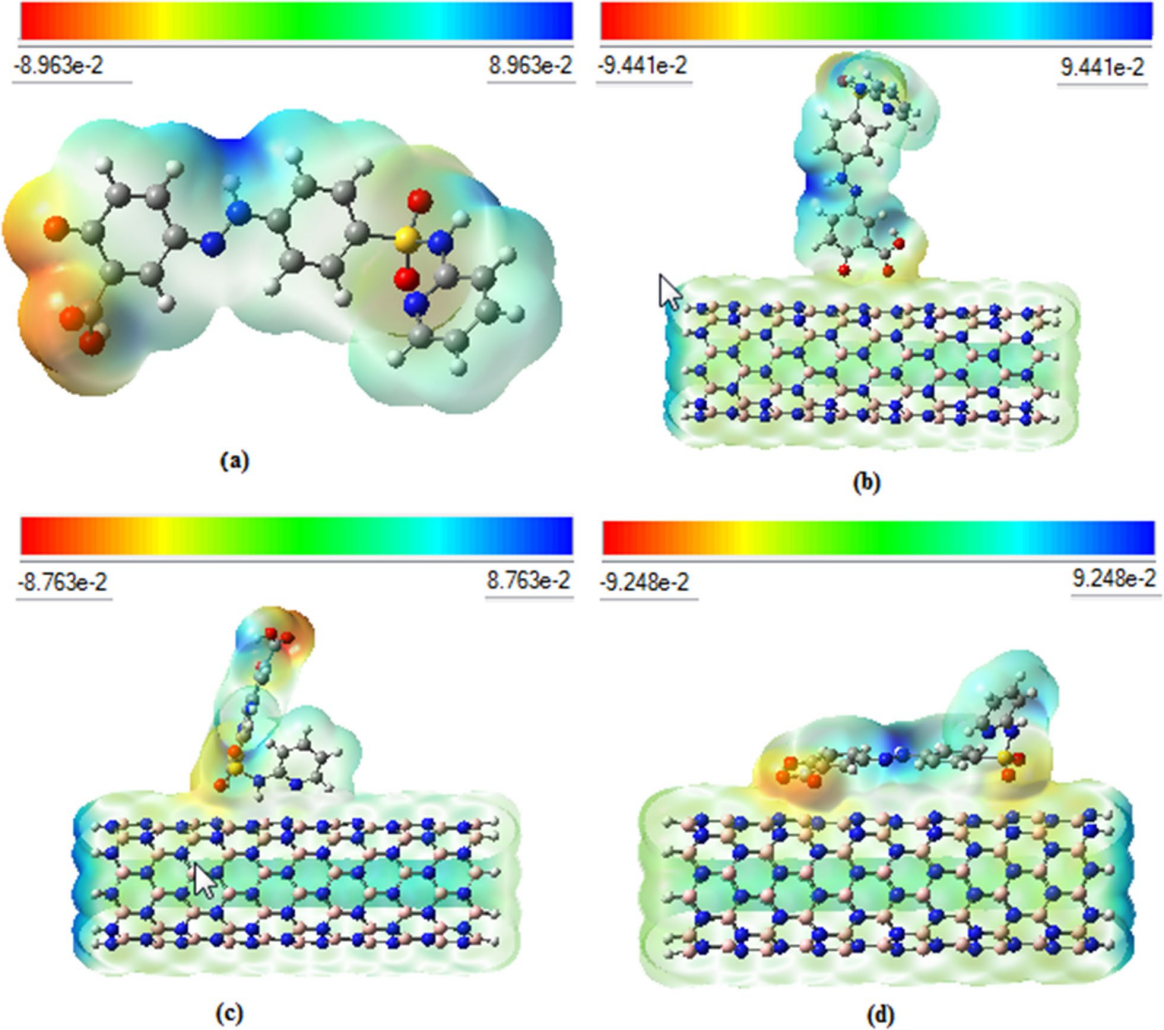
Molecular Electrostatic potential (MEP) surfaces for (**a**) SSZ (**b**) SSZ (keto)–BNNT-via carbonyl (**c**) SSZ (keto)–BNNT-via pyridine (**d**) SSZ (keto)–BNNT-via sulfonamide

These diagrams are suitable for visualizing the reaction sides on the molecular surface as a function of the charge density distribution. Furthermore, MEP and ESP for SSZ-BNNT complexes show the performance of active sites of SSZ after interactions with nano-carrier. The MEP surfaces for SSZ and SSZ-BNNT complexes are indicated in Fig. 4. The potential changes from − 8.963e^−2^ to 8.963e^−2^ a.u. of SSZ has introduced possible sides for nucleophilic attack, as can be seen in Fig. 4, and it has been mentioned before. The MEP color code values between the blue and red for the SSZ-BNNT complexes via carbonyl, pyridine and sulfonamide are range from − 9.441 to 9.441 e^−2^ a.u., − 8.763 to 8.763 e^−2^ a.u. and − 9.248 to 9.248 e^−2^ a.u., respectively.

Moreover, the charge distribution around the carbonyl, sulfonamide and pyridine sites can also be evaluated from their molecular electrostatic potential (MEP) maps. The red color on the O atoms in the most stable state indicates the negative electrostatic potential, which can act as a nucleophilic side in the interaction with BNNT. This evidence arises from the highly electronegative nature of O atoms, and as previously concluded, is the most reactive moiety. The results are compatible with binding energy calculations. The MEP graphs of the complexes show that the electron density of the active sites is reduced compared to the pure SSZ, which indicates the efficiency of the drug–carrier interaction.

The Mulliken atomic charges of selected SSZ atoms in the interaction with BNNT are presented in Table 3. The computed QM atomic charges were applied in calculation of intermolecular electrostatic interactions in further MC simulation computations. Moreover, comparing of SSZ atomic charges before and after the interaction with BNNT indicates the charge transfer between SSZ and BNNT and confirms the interaction. Comparison of the atomic charges of the site of interactions with BNNT specified the effectiveness of that site. As can be seen in Table 3, the atomic charges of SSZ interaction sites have changed after binding with BNNT. For instance, partial atomic charges of S and O in the sulfonamide group of SSZ were 0.90 and − 0.47 before the interactions, which changed to 0.67 and − 0.13 after the interactions with BNNT, respectively. The average atomic charges of (B and N) in the BNNT are (0.83 and − 0.83), and (0.92 and − 0.92) before and after the interaction with drug which approve the charge transfer between drug and nanotube.

**Table 3:** Mulliken atomic charges of SSZ and SSZ-BNNT complexes

Species	C1	C2	O1	O2	O3	N1	N2	N3	N4	S	O	H2	H3
SSZ (enol)	0.2	0.44	− 0.3	− 0.34	− 0.31	− 0.21	− 0.20	− 0.60	− 0.40	0.90	− 0.47	0.25	0.28
SSZ (keto)	0.24	0.41	− 0.28	− 0.3	− 0.28	− 0.32	− 0.18	− 0.60	− 0.41	0.90	− 0.47	0.25	0.28
SSZ (enol)–BNNT-via carbonyl-ver1	0.28	0.45	− 0.61	− 0.57	− 0.40	− 0.30	− 0.29	− 0.57	− 0.57	0.49	− 0.10	0.40	0.38
SSZ (enol)–BNNT-via carbonyl-ver2	0.28	0.45	− 0.60	− 0.57	− 0.40	− 0.30	− 0.29	− 0.57	− 0.57	0.49	− 0.10	0.40	0.38
SSZ (enol)–BNNT-via sulfonamide	0.25	0.46	− 0.57	− 0.39	− 0.57	− 0.31	− 0.29	− 0.60	− 0.57	0.72	− 0.13	0.39	0.38
SSZ (keto)–BNNT-via carbonyl-ver1	0.42	0.69	− 0.45	− 0.54	− 0.42	− 0.61	− 0.33	− 0.74	− 0.57	0.49	− 0.12	0.37	0.36
SSZ (keto)–BNNT-via carbonyl-ver2	0.30	0.48	− 0.43	− 0.59	− 0.37	− 0.6	− 0.22	− 0.56	− 0.57	0.49	− 0.12	0.41	0.38
SSZ (keto)–BNNT-via sulfonamide	0.29	0.52	− 0.52	− 0.60	− 0.43	− 0.6	− 0.20	− 0.60	− 0.56	0.67	− 0.13	0.42	0.38
SSZ (keto)–BNNT-via pyridine	0.27	0.47	− 0.38	− 0.55	− 0.34	− 0.61	− 0.22	− 0.56	− 0.47	0.63	− 0.17	0.37	0.48

The partial charges of the atoms of SSZ-BNNT complexes increase the intermolecular interactions in aqueous solution. Furthermore, as seen in Table 2, the dipole moments of keto SSZ-BNNT complexes are larger than enol SSZ-BNNT. Therefore, it is predicted that the solubility of the keto nanodrug in aqueous solution is higher than that of enol nanodrug, and therefore drug transport in physiological environment is facilitated.

To investigate intermolecular bonding and interaction among bonds the natural bond orbital (NBO) parameters for the interactions of SSZ-BNNT complexes are calculated and the obtained results are tabulated in Table 4. The stabilization energy, E2 (second-order perturbation energy) [88], associated with the donor orbital (i) and acceptor orbital (j), E (j) and E (i) are orbital energies, Fij is the off‒diagonal NBO Fock matrix element j. The value of E (2) illustrates, the nature of interaction between donor orbital electron and acceptor orbital electron, and the greater the extent of conjugation of the whole system. The results of Table 4 indicated that the strongest intermolecular interaction of SSZ-BNNT via carbonyl groups, ver 1&2, occurs through electron transition from LP (O1) → π* (B-N) with E (2) values of 14.55 and 5.92 kcal mol^−1^ respectively. However, the calculated binding energies indicated that SSZ showed the strongest binding with BNNT via carbonyl-ver2 due to the more electron transition between BNNT and SSZ. NBO analysis of the SSZ–BNNT interaction via sulfonamide revealed that transition from LP (O2) → π* (B-N) with E (2) value of 5.57 kcal mol^−1^ was preferred to LP (O (S)) → π* (B-N) with E (2) value of 3.26 kcal mol^−1^. Remarkably, the preferential adsorption of SSZ on the surface of BNNT occurs through carbonyl groups. These results are compatible with calculated binding energies.

**Table 4:** The second-order perturbation energies for SSZ–BNNT intermolecular interactions

Species	Donor	Acceptor	E (2) (kcal/ mol)	E (j)-E (i) (a.u.)	F (i,j) (a.u.)
SSZ (enol)–BNNT-via carbonyl-ver1	LP (O1)	π* (B-N)	14.55	0.78	0.1
	LP (O3)	π* (B-N)	4.06	0.75	0.053
SSZ (keto)–BNNT-via carbonyl-ver2	π (B-N)	σ* (O2-H2)	7.01	0.58	0.061
	π (C1-O1)	π* (B-N)	2.59	0.41	0.031
	LP (O1)	π* (B-N)	5.92	0.75	0.064
	LP (O2)	π* (B-N)	3.01	0.35	0.3
SSZ (keto)–BNNT-via sulfonamide	LP (O (S))	π* (B-N)	3.26	0.34	0.03
	LP (O2)	π* (B-N)	5.57	0.32	0.038
SSZ (keto)–BNNT-via pyridine	LP (O (S))	π* (B-N)	5.76	0.33	0.039
	LP (N4)	π* (B-N)	5.01	0.38	0.065

Interaction of SSZ–BNNT via pyridine arises from the electron transition from LP (N4, pyridine) to π* (B-N).

AIM calculations were also performed in one of the most stable configurations of the system to specify the type of molecular interactions. According to the AIM theory, upon formation of a bond between two neighboring atoms, a critical point appears between them. The nature of the bond is described by the total electronic density, *ρ* (*r*), and its corresponding Laplacian, ∇^2^*ρ (r)* at that point. The Laplacian is the second derivative of a scalar function. Therefore, the Laplacian of electronic density gives information about the tendency of electronic density to concentrate or deplete. The Laplacian of electronic density is related to the energetic topological parameters by the following equation [89]:

5 $$\nabla 2\rho (r)\, = \,2G(r)\, + \,V(r)$$

where *G* (*r*) and *V* (*r*) are the kinetic and potential electronic energy densities at the critical points, respectively. Positive values of Laplacian at the bond critical point (BCP) show that *G* (*r*) is greater than *V* (*r*), which suggests depletion of the electronic charge along the bond path. This is characteristic of closed-shell interactions such as van der Waals interactions or hydrogen bonding. However, negative values of Laplacian show excess potential energy at the BCP, indicating shared interactions such as covalent bonds.

**Fig. 5:**
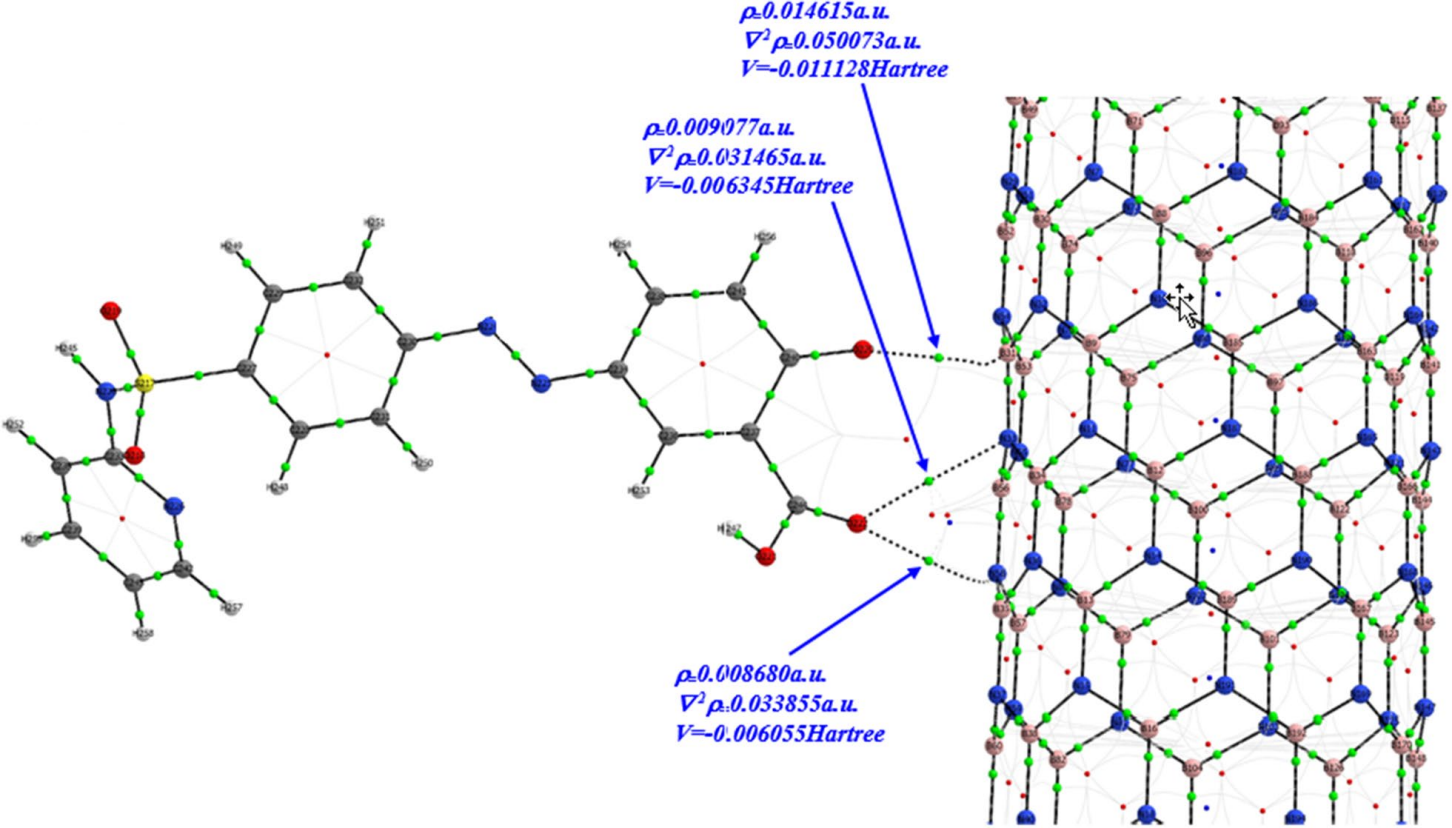
Atoms in molecules (AIM) analysis of the interaction of SSZ–BNNT

The − *G* (*r*)*/V* (*r*) ratio, *GVR*, is also a descriptor for the nature of molecular interactions. For *GVR* > *1*, the intermolecular interaction is non-covalent and for 0.5 < *GVR* < 1 the intermolecular interaction is partly covalent [90]. The results of AIM analysis of the interaction of SSZ and BNNT via carbonyl have been presented in Table 5 and clarified in Fig. 5. The figure indicates three intermolecular bonds between SSZ and BNNT: via carbonyl SSZ and, B and two N atoms in BNNT. As shown in Table 5, the *GVR*s are slightly greater than unity, which also confirms that the intermolecular bonds between SSZ and BNNT are non-covalent.

**Table 5:** Topological parameters (in a.u.) and intermolecular interaction energy (in kcal mol^−1^) for SSZ (keto)–BNNT-via carbonyl at the B3LYP level of theory

Intramolecular bond	$r$(Å)	$\rho (r)$	$\nabla^{2} \rho (r)$	E	G	V	GVR
$C_{240} - O_{220} \cdots B_{31}$	2.55	0.014615	0.050073	3.49	0.011823	− 0.011128	1.062
$C_{244} - O_{222} \cdots N_{58}$	3.14	0.008680	0.033855	1.90	0.007260	− 0.006055	1.199
$C_{244} - O_{222} \cdots N_{33}$	3.01	0.009077	0.031465	1.99	0.007105	− 0.006345	1.120

* According to Fig. 1, C240, O220, C244 and O222 are C_1_, O_1_, C_2_ and O_3_

In the gas phase QM calculations, the molecules were considered as isolated species, and the structure’s stability and binding energies were evaluated. Hydration of drug is vitally important since numerous biological processes occur in physiological media. Therefore, the effect of hydration on the more stable complexes has also been considered by polarizable continuum model (PCM) [91, 92]. In this model, solvent is treated as a polarizable continuum with a dielectric constant, ɛ, instead of explicit solvent molecules. The results of the QM calculations in solution phase are presented in Table 6. As can be seen, the energy values in water media are reduced compared to the gas phase results (Table 2). The electric field applied to the solute by the solvent dipole interacts with the molecular dipole, which leads to net stabilization, and thus increases the stability of the structures in the polar solvent (water) rather than in the gas phase.

**Table 6:** QM results in water medium

Species	Dipole moment (Debye)	Energy (Hartree)	Energy (kcal /mol)	Binding energy (kcal /mol)
BNNT	13.84	− 7901.11	− 4,958,028.55	−
SSZ (keto form)	13.23	− 1687.24	− 1,058,760.03	−
SSZ (keto)–BNNT-via carbonyl-ver1	25.21	− 9588.45	− 6,016,848.33	− 59.75
SSZ (keto)–BNNT-via carbonyl-ver2	30.21	− 9588.45	− 6,016,847.54	− 58.96
SSZ (keto)–BNNT-via sulfonamide	26.59	− 9588.47	− 6,016,860.76	− 72.18
SSZ (keto)–BNNT-via pyridine	17.54	− 9588.46	− 6,016,851.84	− 63.26

The binding energies of SSZ (keto form) and BNNT via carbonyl-ver1&2, sulfonamide and pyridine in water media were − 59.75, − 58.96, − 72.18 and − 63.26 kcal/mol respectively. The results of QM water phase confirm the possible interactions between SSZ drug and BNNT carrier in solution.

Although, a drug–water potential energy surface cannot be constructed from accurate QM calculations, even with recent growth in computer power, because too many points are required. While the study of the interactions in aqueous solution by QM method is performed using the PCM model for the solution phase, it could not use all the solvent molecules in calculations. Clearly, the construction of a potential energy surface for such large systems, using more elaborate QM techniques, is not currently feasible. Thus, it is suitable to use computer simulation methods for these systems. The ability to accurately calculate solvation free energies and association free energies in solvent using molecular simulation methods is an important development in computational chemistry. These methods are widely used not only in studies of solvation free energies, but also in studies of binding free energies.

In the next part, solvation behavior of these nano drug complexes is investigated, and the possibility of the interaction between SSZ and BNNT in aqueous solution is evaluated by MC simulation.

### MC

Many chemical reactions occur in liquid solutions, and water plays an important role in various molecular processes in chemistry and biochemistry. The solute–solvent interactions are responsible for the significant changes in chemical and physical characteristics of the solute in transition from gas phase to solvent phase. Investigation of the structural, electronic, and optical properties of 4-(dicyanomethylene)-2-tert-butyl-6-(1,1,7,7-tetramethyljulolidin-4-yl-vinyl)-4H-pyran (DCJTB) semiconductor dye by performing both experiments and DFT calculations [93] has indicated that the solvents have a significant effect on the properties of DCJTB organic solutions and films. Study of the structural, electronic, spectroscopic and optical properties of N,N’-Dipentyl-3,4,9,10-perylenedicarboximide (PTCDI-C5) small molecule [94] has also specified the performance of the solvent effect on the structural parameters, refractive index, and the electronic band gap structure. In Another study, 1,4-Bis [2-(3-N-ethylcarbazoryl)-vinyl]benzene (BCzVB) in different solvents have been performed to explore its electronic structure and photo-physical properties using quantum chemistry calculations [95]. The results have confirmed that the solvent environment enhances characteristic properties of the BCzVB.

**Fig. 6:**
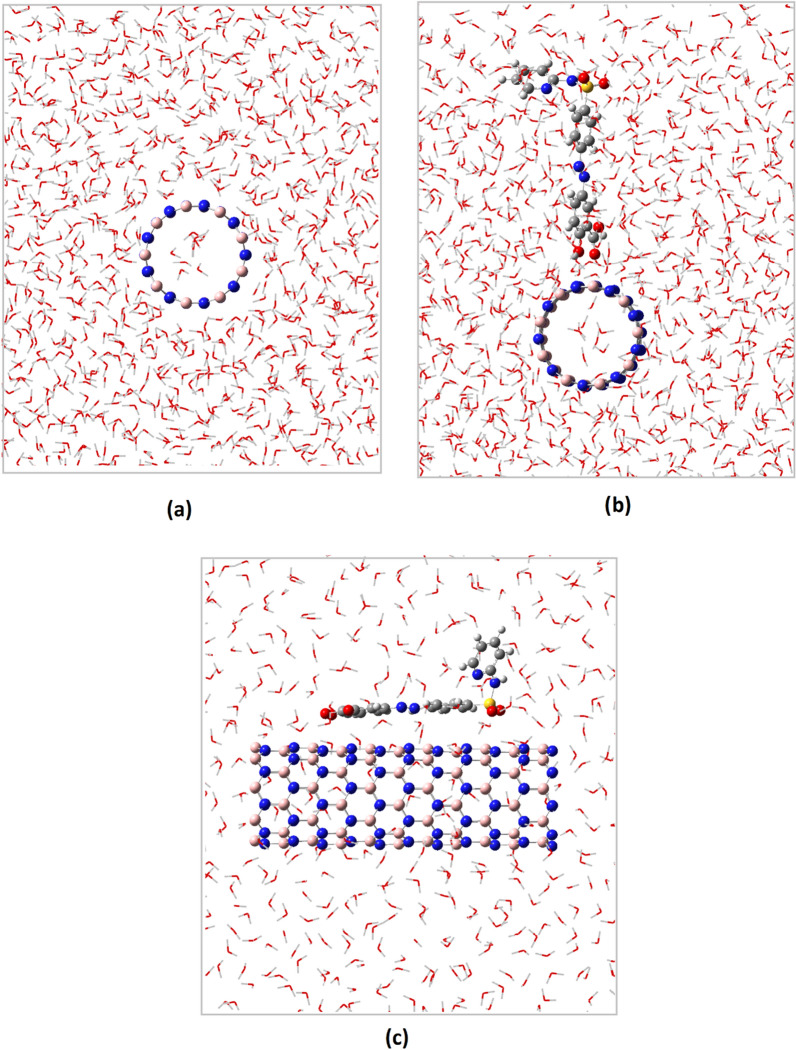
Snapshot of the simulation of **a** BNNT-top view, **b** SSZ-BNNT (carbonyl)-top view and **c** SSZ-BNNT (sulfonamide)—side view in aqueous solution

In QM part of our study, probability of the interaction of SSZ and BNNT was studied. Then the interactions in solution were evaluated by MC simulation. First, the total interaction energies of the structures (BNNT, SSZ and SSZ-BNNT complexes) were determined using MC simulations to evaluate the interaction of SSZ and BNNT in aqueous solution. Snapshot of configuration that extracted from MC simulation of BNNT and SSZ-BNNT in aqueous solution is shown in Fig. 6. The figure presents qualitative scheme of the hydration shells surrounding the BNNT.

The total energy (E_tot_) in solution is explained by the sum of contributions of solute–water, water–water and intramolecular interaction energies. The energies obtained from the MC simulation are presented in Table 7. This table includes the number of water molecules in the box (N_H2O_), the energy contributions of the solute–H2O interaction energy (E_soln_) and, the electrostatic contribution and the van der Waals contribution to the solute–H2O interaction energy.

MC simulations results showed that the total energy of SSZ enol and keto were − 11.36 and − 30.85 kcal/mol respectively which was lower than that of BNNT-SSZ complexes. Total energies of BNNT-SSZ (keto) complexes in solution were larger than BNNT-SSZ (enol) complexes.

The interaction of SSZ and BNNT increases the electrostatic contribution to the solute-H2O interaction energy, and then the total interaction energy of SSZ also increases. Indeed, the interaction of SSZ with nanotube enhances partial atomic charges, thereby increasing the electrostatic contribution to the SSZ–H2O interaction energy. The electrostatic contribution to the BNNT–SSZ interaction energy is larger than the van der Waals contribution. The partial atomic charges can directly affect the electrostatic terms of intermolecular energies. In aqueous solution of BNNT and SSZ-BNNT complexes, electrostatic interactions play the main role in intermolecular interactions. On the other hand, the role of Van der Waals interactions in the intermolecular interactions of BNNT and BNNT-SSZ solutions is almost the same. Therefore, it can be concluded that the van der Waals interactions do not affect the intermolecular energies after the interaction of SSZ and BNNT in water. These results are consistent with our previous research [43]. A simulation study of cisplatin encapsulated in nanotubes has also showed that the electrostatic interaction energies were increased after interaction of drug and BNNT, while Van der Waals energies did not change [43]. Encapsulation of alkylating anticancer drug into CNT have also increased the electrostatic as well as total interaction energies, but did not affect the Van der Waals interactions in solution [33]

Solubility improvement of SSZ from its dosage form is an important subject for its in vivo bioavailability and therapeutic efficiency. Therefore, enhancement of the solubility of SSZ was the aim of our research. Solvation free energies of BNNT, SSZ (keto and enol) and SSZ-BNNT complexes were obtained by Eq. 2. Computed solvation free energies were accessible in Table 8. As it is present, the solvation free energy of SSZ was considerably enhanced after interaction with BNNT.

**Table 7:** MC simulation results (energies are in kcal /mol)

Species	N_H2O_	E	E_soln_	Electrostatic contribution in E_soln_	van der Waals contribution in E_soln_
BNNT	3990	− 255.66	− 290.34	− 291.47	1.12
SSZ (enol form)	4059	− 11.36	− 3.30	− 3.29	− 0.01
SSZ (keto form)	4059	− 30.85	− 22.92	− 22.88	− 0.04
SSZ (enol)–BNNT-via carbonyl-ver1	3983	− 230.89	− 265.95	− 267.11	1.16
SSZ (enol)–BNNT-via carbonyl-ver2	3984	− 231.31	− 266.30	− 267.46	1.15
SSZ (enol)–BNNT-via sulfonamide	3980	− 227.90	− 261.90	− 263.23	1.33
SSZ (keto)–BNNT-via carbonyl-ver1	3958	− 241.91	− 275.87	− 277.07	1.20
SSZ (keto)–BNNT-via carbonyl-ver2	3970	− 233.42	− 267.83	− 269.10	1.27
SSZ (keto)–BNNT-via sulfonamide	3977	− 230.27	− 264.30	− 265.57	1.27
SSZ (keto)–BNNT-via pyridine	3964	− 214.66	− 245.77	− 246.683	0.908

BNNT is a full of charge system and so its contribution of electrostatic interactions in solute–solvent interactions is significant (-291.47 k cal mol^−1^). Therefore, the solvation free energies of BNNT-SSZ complexes are expected to be significant. While solvation free energy of SSZ was about − 21 kcal mol^−1^ due to small electrostatic contribution in drug–solvent interaction energy (− 3.29 and − 22.88 kcal mol^−1^ for enol and keto form respectively). Therefore, it can be predicted that the solvation free energies should be of the same order. When SSZ interacts with BNNT, the resulting complexes should be more stable in water than the drug to increase the probability of reaction in aqueous environment.

To predict the probability of interaction of BNNT and SSZ in aqueous environment, the association free energies were also calculated. As can be seen in Table 8, Association free energy of the interaction of BNNT and SSZ via all sites is negative. Thus, this drug can interact with BNNT in aqueous solution. It should be noted that the main contribution to solute-H2O interactions is related to the electrostatic energy. Electrostatic interaction energies of all SSZ-BNNT complexes in H2O are almost identical (see Table 7) and higher than SSZ itself. Thus, SSZ can interact with BNNT due to appropriate solvation free energy and association free energy.

**Table 8:** Solvation free energies (ΔG_sol_) and association free energies (ΔG_ass_) of drugs–CNT compounds

Species	ΔG_sol_ (kcal /mol)	ΔG_ass_ (kcal /mol)
BNNT	− 248.2917	
SSZ (enol form)	− 21.134	
SSZ (keto form)	− 21.7606	
SSZ (enol)–BNNT-via carbonyl-ver1	− 223.0949	− 46.3308
SSZ (enol)–BNNT-via carbonyl-ver2	− 223.4236	− 46.0021
SSZ (enol)–BNNT-via sulfonamide	− 220.2629	− 49.1628
SSZ (keto)–BNNT-via carbonyl-ver1	234.464	− 35.5883
SSZ (keto)–BNNT-via carbonyl-ver2	− 225.7073	− 44.345
SSZ (keto)–BNNT-via sulfonamide	− 222.5613	− 47.491
SSZ (keto)–BNNT-via pyridine	− 206.97	− 63.08

To estimate distribution of water molecules inside and outside the BNNT, radial distribution functions (RDFs) were also computed.

RDF is a feature that defines the structure of the fluids. It is determined as the ratio of the local density ($\rho (r)$) to the bulk density. RDF is defined as a function of the distance from the central axis of the nanotube, $g\left(r\right),$

6 $$g\left(r\right)=\frac{\rho \left(r\right)}{{\rho }_{bulk}}$$

**Fig. 7:**
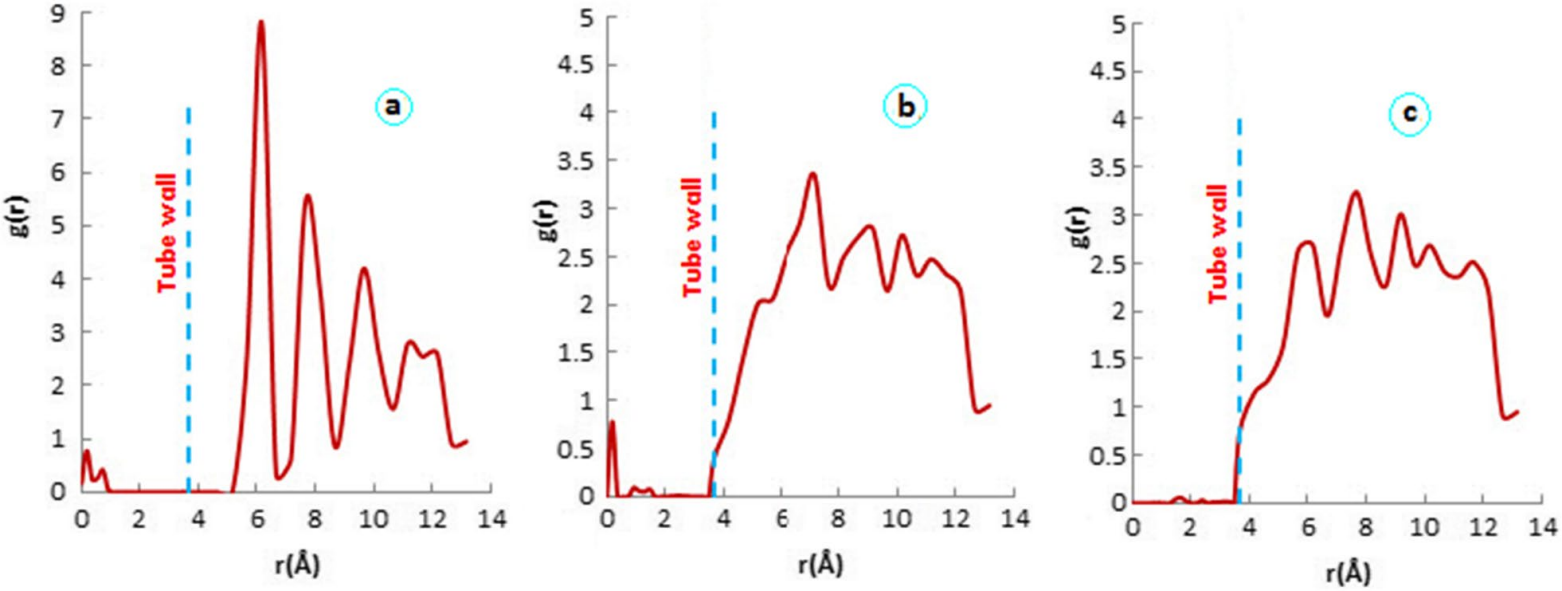
RDFs of water molecules from center of nanotube. **a** BNNT **b** SSZ–BNNT-via sulfonamide **c** SSZ–BNNT-via carbonyl

The RDF diagrams for the BNNT and two complexes of SSZ with BNNT via sulfonamide and carbonyl were depicted in Fig. 7. The diagrams indicate g (r) of water molecules inside and outside the BNNT versus r (which is the distance from the axis of the nanotube). The inner and outer plots are separated by a dashed line located at 3.67 Å (BNNT radius). The inner peaks of the RDF diagrams are mainly located in the center of the nanotube (0–1 Å from the central axis) and their intensity is much lower than the outer ones.

As can be seen, the outside RDF graphs (r > 3.67 Å) have peaks that correspond to the formation of shell-like water molecules around the BNNT surface.

In pure BNNT’ RDF graph (Fig. 7a), the first and highest peak is at 6 Å (2.33 from the outer surface of nanotube) and the next layers with less intensity are located around 8, 10 and 12 Å. However, as seen in Fig. 7b, c for BNNT-SSZ complexes, the intensity of all peaks are reduced and broadened due to the interaction of water molecules with each other and with the drug molecule that binds to the surface of the nanotube. In fact, the atomic charges due to interaction of SSZ with BNNT caused water molecules within the shells to point to each other and produce a broader peak than pure BNNT. The intensity of the first RDF peak (6 Å) of SSZ–BNNT-via sulfonamide (Fig. 7b) is less than that of SSZ–BNNT-via carbonyl (Fig. 7c). This is due to the direction of the adsorbed drug in SSZ–BNNT-via sulfonamide complex, which is parallel to the nanotube surface, and therefore, the density of water molecules in that distance is reduced. As seen in Fig. 7c, the intensities of all peaks are almost the same due to the vertical orientation of the SSZ molecule to the surface and the interaction of water molecules with the drug.

As it is seen, all RDF plots show limit of bulk water at distance greater than 9 Å from the surface that is consistent with our previous researches [33, 43]

### MD

In order to investigate the dynamics of the interaction of SSZ (keto)-BNNT from different sites individual MD simulations for SSZ (keto)-BNNT systems were performed. Starting atomic coordinate obtained from optimized QM structures. Then initial structures were equilibrated via MD simulation methods.

To evaluate the dynamics of the interactions, the contributions of different energies of the system during the simulation were calculated and listed in Table 9. According to the results, the change of total potential energy was mainly due to the change of non-bonded energy. As can be seen, during the simulation, bonding energies did not change considerably. This means that the conformation of the structures has not changed significantly. However due to the interactions in aqueous environment, the non-bonding energies (which are electrostatic and van der Waals interactions, Table 7) changed considerably and caused a drastic decrease in the potential energy. In fact, during the interactions, the contact area between SSZ and BNNT was increasing, which could increase the van der Waals and electrostatic interactions and reduce the total potential energy. Finally, the potential energy of the system underwent a substantial decrease during the simulation time, which indicated that the whole system had reached an equilibrium state due to the tendency to achieve a more stable energetic state.

**Table 9:** Energy details for initial and final structures of considered SSZ-BNNT systems through MD simulation (in kcal mol^1^)

Energy (kcal mol^−1^)	ΔE = E (Final configuration)-E (Initial configuration)			
	SSZ (keto)–BNNT-via carbonyl-ver1	SSZ (keto)–BNNT-via carbonyl-ver2	SSZ (keto)–BNNT-via sulfonamide	SSZ (keto)–BNNT-via pyridine
Total energy	− 1527.21	− 1522.57	− 1709.82	− 1538.69
potential energy	− 1539.9	− 1531.43	− 1701.12	− 1545.61
Bond	− 2.74	− 4.14	− 73.05	− 8.24
Angle	− 81.86	− 5.75	− 184.27	− 9.24
Torsion	− 9.91	− 1.04	− 17.75	− 6.68

**Fig. 8:**
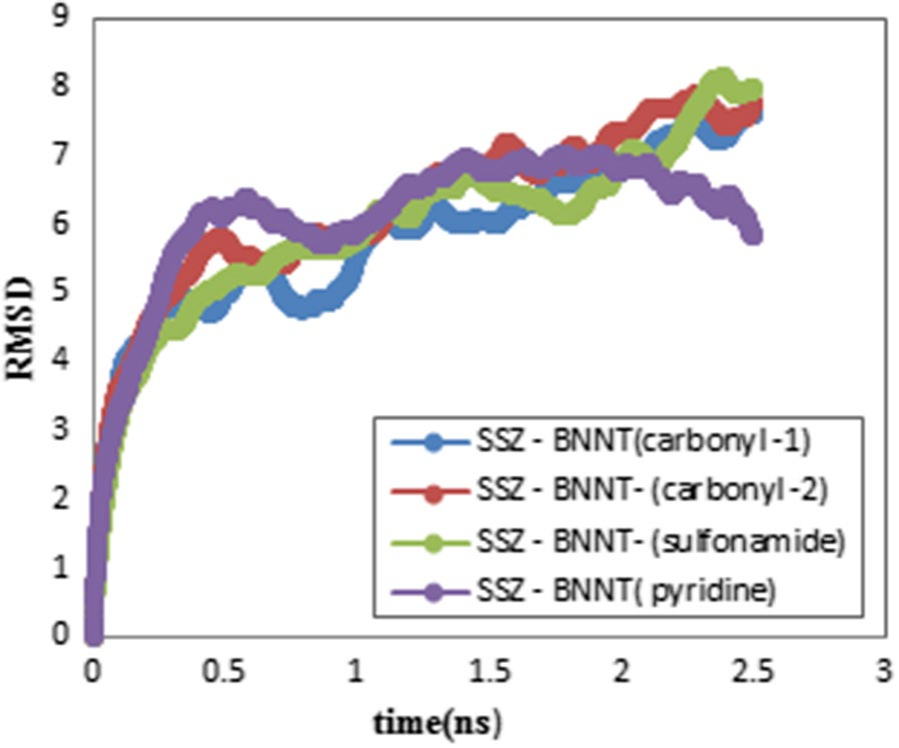
RMSD diagrams for SSZ (keto)–BNNT interactions

Figure 8 shows root-mean-square deviation (RMSD) for the selected systems to determine the equilibrium state of the studied systems. The results show that the system has reached the equilibrium state after 1 ns. However, it can be concluded from Fig. 8 that interaction of SSZ-BNNT through carbonyl group has reached the equilibrium faster than the other two complexes.

## Conclusions

The solubility performance of drugs is one of the most challenging features in medicinal chemistry. Therefore, enhancement of the solubility of sulfasalazine in aqueous environment was planned in this research. In the first section, binding energies of two tautomers of SSZ (enol and keto forms) with (9,0) BNNT were calculated applying density functional calculations. Among the three possible interactions of SSZ (from pyridine ring, sulfonamide, and carbonyl group) with BNNT, the interaction of keto form via carbonyl was preferred.

Comparing the SSZ atomic charges before and after interactions with BNNT showed charge transfer between SSZ and BNNT. To investigate intermolecular bonding and interaction among bonds, NBO analysis was applied for the interactions of SSZ-BNNT complexes. The strongest SSZ-BNNT intermolecular interaction occurred via carbonyl groups through electron transition from LP (O1) → π* (B-N), while in the interactions via sulfonamide and pyridine, LP (O2) → π* (B-N) and LP (N4) → π* (B-N) transitions were preferred, respectively. The AIM analysis of the interaction of SSZ and BNNT via carbonyl confirmed that the intermolecular bonds between SSZ and BNNT are non-covalent.

This evidence is compatible with the binding energy calculations.

In the second section, stability of drug–BNNT complexes was estimated by Monte Carlo simulation and free energy perturbation method. Computed association free energies confirm possibility of the interaction of BNNT and SSZ in aqueous solution.

To investigate the dynamics of the interaction of SSZ (keto)-BNNT from different sites, MD simulation was performed. The results indicated that bonding energies did not change considerably, and therefore, the conformation of the structures did not change significantly during the simulation. ESP calculations evaluated the performance of SSZ active sites, and confirmed that carbonyl groups can act as a nucleophilic side interacting with the nano-carrier.

Computations indicated that the solvation free energies of all SSZ-BNNT complexes in water were significantly larger than SSZ alone. Therefore, BNNT held great potential to act as a carrier for SSZ.

Our main result can be concise as: interaction of SSZ with BNNT, improved solubility of SSZ in biological fluids. Therefore, the use of BNNT as a drug delivery system for SSZ has been proposed to enhance the bioavailability, reduce drug dosage and side effects.

### Supplementary Information

**Additional file 1: Table S1.** Selected bond lengths (in Å) of SSZ before and after the interaction with BNNT.

## Data Availability

The optimized geometries of the molecules, and datasets generated and analyzed during the current study are available from the corresponding author upon request.
