# Supplementary material for: Interaction of sulfasalazine with outer surface of boron-nitride nanotube as a drug carrier in aqueous solution: insights from quantum mechanics and Monte Carlo simulation
Source: BMC Chem. 2023 Nov 28;17(1):169. doi: 10.1186/s13065-023-01088-w (PMC10683185; doi:10.1186/s13065-023-01088-w)
Supplement: Supplementary file 1 — Additional file 1: Table S1. Selected bond lengths (in Å) of SSZ before and after the interaction with BNNT. [file 13065_2023_1088_MOESM1_ESM.pdf]

Table S1- selected bond lengths (in Å) of SSZ before and after the interaction with BNNT

**Species**	**C1-O1**	**O1-H1**	**C2-O2**	**C2-O3**	**O2-H2**	**N1-N2**	**S-O**	**N3-H3**
SSZ (enol)	1.3464	0.9641	1.3623	1.2046	0.9677	1.2553	1.46	1.0116
SSZ (keto)	1.2201	-	1.3637	1.1946	0.9645	1.3157	1.4525	1.0118
SSZ (enol)-BNNT-via carbonyl -ver1	1.3667	0.9955	1.3827	1.2318	0.9961	1.2924	1.4403	1.0204
SSZ (enol)-BNNT-via carbonyl -ver2	1.3692	0.9935	1.3831	1.2321	0.9958	1.2923	1.4404	1.0205
SSZ (enol)-BNNT-via sulfonamide	1.3689	0.9932	1.3937	1.2272	0.9962	1.2958	1.4315	1.0204
SSZ (keto)-BNNT-via carbonyl -ver1	1.2482	-	1.3905	1.2222	0.9936	1.3428	1.4402	1.0206
SSZ (keto)-BNNT-via carbonyl -ver2	1.2563	-	1.3872	1.2256	0.9870	1.3349	1.4441	1.0122
SSZ (keto)-BNNT-via sulfonamide	1.2724	-	1.3658	1.2293	1.0347	1.3385	1.4392	1.0207
SSZ (keto)-BNNT-via pyridine	1.2439	-	1.3999	1.2171	0.9936	1.3491	1.4304	1.0352
